# Poster Session I - A35 INVESTIGATING THE STING MEDIATED RESPONSE TO ENTERIC ADENOVIRUS INFECTION IN THE HUMAN INTESTINAL EPITHELIUM.

**DOI:** 10.1093/jcag/gwaf042.035

**Published:** 2026-02-13

**Authors:** E Karam, S Girardin

**Affiliations:** Laboratory Medicine and Pathobiology, University of Toronto, Toronto, ON, Canada; Laboratory Medicine and Pathobiology, University of Toronto, Toronto, ON, Canada

## Abstract

**Background:**

Dysregulated STING signalling in intestinal and colonic tissue is associated with dysbiosis and increased susceptibility to enteric pathogens, however, the correlation between the cGAS-STING pathway and IBD appears to be context dependant and requires further investigation. Human adenovirus are non-enveloped dsDNA viruses classified into 7 distinct species. Species F includes both type 40 (Ad40) and type 41 (Ad41); enteric pathogens that specifically infect the intestinal epithelium and are the third leading cause of infantile gastroenteritis. Adenovirus infection has been shown to disrupt the host microbiome disrupting intestinal homeostasis, however, research into the association of viral infection and STING activation in the intestinal epithelium remains poorly characterized.

**Aims:**

I hypothesize that epithelial intrinsic STING signalling serves a critically protective role in the host defence response, with dysregulation causing increased susceptibility to viral infection. Aim 1 is to characterize the physiological effect of epithelial intrinsic STING signalling to intestinal homeostasis. Aim 2 is to then investigate the implications of the STING-mediated response to enteric adenovirus infection.

**Methods:**

Through our existing collaboration with the Canadian National Organoid Network (CNON) and existing library of patient-derived organoid samples, I utilize ileal and duodenal organoids to serve as a primary human model. To elucidate if the cGAS-STING pathway serves as the primary line of defence against enteric adenovirus, I infected human duodenal and ileal organoids with Ad41 to study the host response to infection through western blot, RT-qPCR, and confocal microscopy.

**Results:**

Human duodenal organoids possess a functional cGAS-STING pathway, activated by diABZI. Despite robust STING activation by agonists, infection of organoid with Ad41 did not trigger STING phosphorylation, even at varying time points or high viral doses. Infection was confirmed by expression of viral transcripts, suggesting Ad41 evades STING-mediated antiviral signaling in intestinal epithelial cells.

**Conclusions:**

Previous studies on adenovirus sensing by cGAS-STING have been limited to cancer or immune cells infected with the respiratory Ad5 strain. My findings indicate that enteric adenovirus evades cGAS-STING detection in human intestinal epithelium, revealing its ability to exploit this tissue as a replicative niche.

**Funding Agencies:**

CCC, CIHRNew Frontiers in Research Fund

